# Preclinical comparison of prolgolimab, pembrolizumab and nivolumab

**DOI:** 10.1038/s41598-024-72118-3

**Published:** 2024-10-04

**Authors:** Aleksandr Gordeev, Andrei Vaal, Maria Puchkova, Iana Smirnova, Aleksandr Doronin, Anna Znobishcheva, Daria Zhmudanova, Aleksei Aleksandrov, Mikhail Sukchev, Evgeny Imyanitov, Valery Solovyev, Pavel Iakovlev

**Affiliations:** 1 JSC BIOCAD, St.-Petersburg, Russia; 2 Department of Tumor Growth Biology, N.N. Petrov Institute of Oncology, St.-Petersburg, Russia

**Keywords:** Cancer immunotherapy, Drug development

## Abstract

Prolgolimab is a recombinant IgG1-based anti-PD-1 antibody, whose properties were improved by the introduction of the LALA mutation, and which has demonstrated high efficacy in patients with metastatic melanoma. This paper presents the results of comparative preclinical studies of antigen-binding and effector functions involving prolgolimab and conventional IgG4 antibodies, nivolumab and pembrolizumab. None of the studied antibodies had undesirable antibody-dependent cellular cytotoxicity activity. Prolgolimab has shown higher PD-1 receptor occupancy and T-cell activation, but lower propensity to activate antibody-dependent cellular phagocytosis as compared to nivolumab and pembrolizumab. An in vivo study in mice inoculated with CT26.wt cancer cells showed that tumor growth inhibition was 16% for pembrolizumab and 56% for prolgolimab. This study warrants clinical comparison of IgG1- and IgG4-based anti-PD-1 antibodies.

## Introduction

Immune checkpoints are involved in many signaling pathways that control the immune system to ensure “self-tolerance” and regulation of the normal immune response. Programmed cell death protein 1 (PD-1), a receptor localized predominantly on T-lymphocytes, is a central component of this signaling cascade. Interaction between the PD-1 ligand (PD-L1) and PD-1 prevents activation of T-lymphocytes. This downregulation is essential for avoiding autoimmune reactions, although it also suppresses an antitumor immune response^1^. Therapeutic antibodies developed against immune checkpoints, for example, antagonists of PD-1 (pembrolizumab, nivolumab, prolgolimab) and its ligand PD-L1 (atezolizumab, durvalumab, avelumab), have demonstrated high clinical efficacy in a wide spectrum of malignancies^1–3^.

The structure of antibodies, for example, IgG-based therapeutic antibodies, includes variable and constant components, which perform different functions in the immune response. The specificity of target binding (e.g., to the PD-1 receptor) is mediated by the variable domain and it is crucial for the therapeutic effect of these antibodies. However, there is growing evidence that the constant domain, which mediates downstream functions, also plays an important role in this process. For example, the interaction between the Fc fragment and the Fcγ-receptor (FcγR) determines the effector functions of the antibody, such as antibody-dependent cellular cytotoxicity (ADCC), antibody-dependent cellular phagocytosis (ADCP) and induction of cytokine secretion. Increased affinity of the antibody to the FcγRIIIa receptor may cause ADCC-mediated elimination of activated T-cells, while increased affinity to FcγRIa, FcγRIIa and FcγRIIIa may result in ADCP of activated T-cells . The interaction between the Fc fragment and C1q determines complement-dependent cellular cytotoxicity (CDC)^4,5^.

The effector functions of antibodies play a key role in the interaction of antigen–antibody complexes with the immune system. This mechanism is necessary for the elimination of antigen-bearing cells, for example, tumor cells, by NK cells or macrophages. However, the effector functions may negatively affect the efficacy of antitumor therapy by eliminating PD-1^+^ T-cells via ADCC, ADCP and CDC^4^. Consequently, significant efforts are invested in minimization of the effector functions of clinically used antibody-based drugs^4,6,7^.

Currently available therapeutic IgG-based antibodies belong to IgG1, IgG2, or IgG4 isotypes. Each IgG isotype has different affinity of binding to various FcyR that also have different expression patterns on the immune cells^8^. In general, IgG1 isotype elicits high ADCC and CDC, while IgG2 and IgG4 isotypes have low ability to bind FcyR and C1q, resulting in relatively low effector functions^9^.

There are two most commonly employed anti-PD-1 drugs, nivolumab and pembrolizumab, which are both IgG4 humanized antibodies. The choice of this antibody isotype is associated with its relatively low ability to induce effector functions. Published study results indicate a potential decrease in the antitumor effect, which the authors attribute to the binding of IgG4 antibodies to FcγRIa sufficient for ADCP, leading to T-cell elimination^9–14^. There are continuing efforts aimed at development of anti-PD-1 antibodies with reduced effector functions, e.g., by changing their interaction with Fcγ-receptors.

Prolgolimab is a new monoclonal antibody, which has recently been approved for the treatment of metastatic melanoma. This recombinant IgG1 antibody carries a LALA (L234A/L235A) mutation, which reduces its binding to FcγR and has been described as one of the most potent modification reducing the antibody effector functions^15,16^. Consequently, IgG1 antibodies with the LALA mutation may have some advantages over IgG4-based drugs, as the latter still have some residual effector functions.

Although the differences in the efficacy and safety of IgG1 and IgG4 antibodies have been indirectly compared in some clinical studies^16–18^, no direct comparison of their effector functions has been performed so far. The aim of this study was to compare the functions of nivolumab, pembrolizumab, and prolgolimab, associated with both the antigen-binding and constant parts of these antibodies.

## Results

### In silico comparison of PD-1 binding epitopes for nivolumab, pembrolizumab and prolgolimab

The principal mechanism of action of therapeutic anti-PD-1 antibodies is the competition with PD-L1 for binding to PD-1. This interaction is mediated by the variable antigen-binding region of the antibodies. It seems reasonable that the greater this overlap, the higher the potential efficiency of the antibody. Therefore, we analyzed in silico the extent of the PD-L1 interaction surface covered by nivolumab, pembrolizumab and prolgolimab.

Structures of PD-1 complexes in conjunction with prolgolimab, nivolumab, as well as PD-L1 have been published in publicly accessible databases such as RCSB Protein DataBank. These structures, following completion of missing atoms and relaxation through molecular dynamics methods, are suitable for in silico interaction analysis. During the development of prolgolimab, precise in silico models representing its interaction with PD-1 were generated. The accuracy of these models was validated using kinetic tests and further employed for rational design of mutations that optimize binder affinities. As such, we deemed our final binder in silico model suitable for comparative assessments, along with the available crystal structures of other complexes. However, it is essential to emphasize that the validity of all conclusions obtained depends on the validity of this model.

**Fig. 1:**
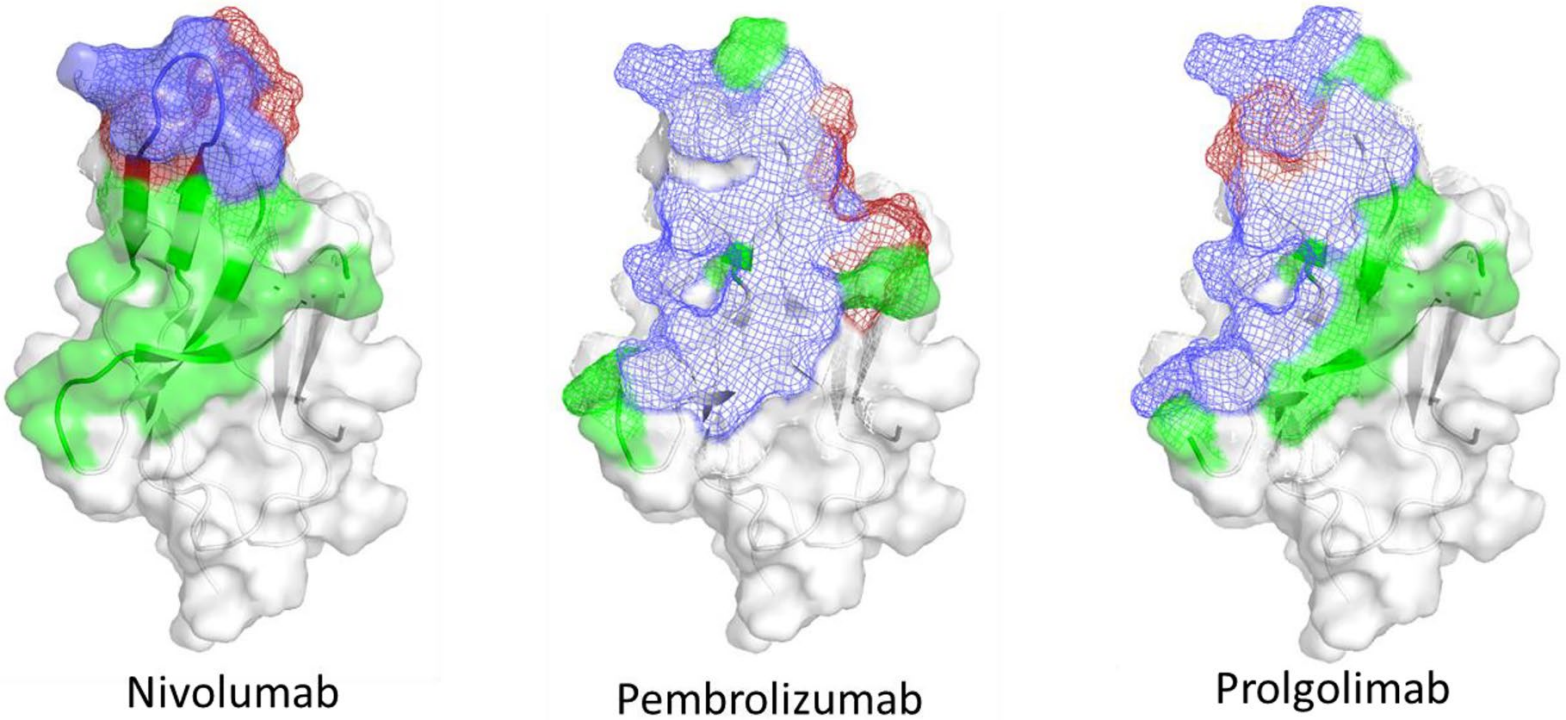
PD-1 binding epitopes of therapeutic anti-PD-1 antibodies and PD-L1 ligand. Blue color marks the overlap between the PD-L1- and antibody-binding epitopes in the PD-1. The green part of the PD-L1 epitope does not overlap with the therapeutic antibody. The red part of the antibody epitope does not overlap with the epitope for PD-L1 binding.

The analysis of the structure of the complex of human PD-1 and its ligand PD-L1 (PDBID 4ZQK, X-ray diffraction) shows that the epitope for PD-1 binding with PD-L1 includes the amino acids V63, N65, Y67, S72-K77, E83, G123, I125, L127-A131, I133, and E135^19^. The same analysis of nivolumab and pembrolizumab in complex with PD-1 (PDB ID: 5WT9 and 5B8C, X-ray diffraction) showed that the epitope for PD-1 interaction consists of T58-S61, S126-Q132 for nivolumab, S62, V63, N65, Y67, S74-K77, I125, L127, A128, K130, A131, I133 for pembrolizumab, and Y67, N73-T75, I125-L127, P129-E135 for prolgolimab^20,21^. The visual comparison of antibodies’ epitopes with the PD-L1 epitope on the PD-1 surface is shown in Fig. 1.

Nivolumab is capable of occupying only a small part of the PD-L1 binding epitope in the PD-1. In contrast, both pembrolizumab and prolgolimab have a potential capacity to cover all surface involved in the interaction between PD-1 and PD-L1.

Prolgolimab is predicted to interact with K134 and E135 of PD-1, while pembrolizumab does not. As highly charged amino acids they contribute a lot into the electrostatic component of the complex enthalpy energy, that is known to be valuable for intermolecular interactions. The real value of these amino acids for PD-1/PD-L1 complex is unknown, but molecular simulations let us assume them potent for the PD-1/PD-L1 complex organization. This makes them important to cover by the interaction inhibitor antibody.

### Nivolumab, pembrolizumab and prolgolimab have similar binding affinity for PD-1

In addition to the binding epitope, the efficacy of an antibody may depend on the affinity of its binding to the antigen. The binding constants of nivolumab, pembrolizumab and prolgolimab with PD-1 were compared by SPR. The mean values of the equilibrium binding constants are shown in Table 1. No significant difference was found in the affinity of PD-1 binding of the tested antibodies.

**Table 1:** Binding constants of nivolumab, pembrolizumab and prolgolimab towards PD-1 receptor, measured by SPR. PD-1 was immobilized on a chip, anti-PD-1 antibodies were in solution. k_a_—association constant. k_d_—dissociation constant. K_D_—equilibrium constant.

anti-PD-1 antibody	k_a_, (M × s)^-1^		k_d_, s^−1^		K_D_, M	
	Mean	SD	Mean	SD	Mean	SD
Nivolumab	2.66 × 10^6^	7.25 × 10^5^	1.83 × 10^–5^	4.66 × 10^–06^	6.89 × 10^–12^	3.16 × 10^–13^
Pembrolizumab	3.73 × 10^6^	2.05 × 10^5^	1.75 × 10^–5^	4.60 × 10^–06^	4.70 × 10^–12^	6.20 × 10^–13^
Prolgolimab	1.76 × 10^6^	5.00 × 10^4^	1.31 × 10^–5^	6.20 × 10^–06^	7.44 × 10^–12^	5.85 × 10^–13^

### T-lymphocyte PD-1 occupancy by nivolumab, pembrolizumab and prolgolimab

We further evaluated the fraction of non-bound PD-1 in the presence of therapeutic antibodies. We incubated PBMCs with 20 μg/mL of nivolumab, pembrolizumab or prolgolimab, and investigated whether the cells were still able to interact with diagnostic fluorescent-labeled anti-PD-1 antibody (clone PD1.3.1.3). All three therapeutic antibodies prevented staining of the cells with the aforementioned reagent thus indicating a complete occupancy of the receptor (Supplementary fig. S1).

We then saturated PBMCs with nivolumab, pembrolizumab or prolgolimab, washed off the unbound antibodies and incubated the cells in medium for 24 h, followed by staining with PD1.3.1.3 and anti-human IgG Fc antibodies.

**Fig. 2:**
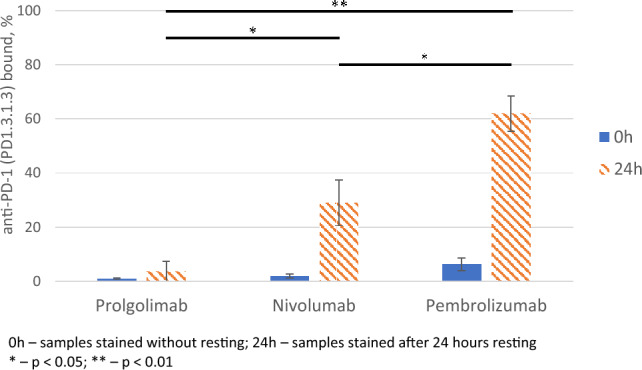
PD-1 receptor occupancy by prolgolimab, nivolumab and pembrolizumab before and after 24-h resting. PBMCs were preincubated with prolgolimab, nivolumab and pembrolizumab, then unbound antibodies were washed off. One portion of the cells was stained with the antibody panel immediately (0h), while the remaining portion was stained after 24 h of the incubation (24 h) in the culture medium. The proportion of T-lymphocytes (CD45^+^/CD3^+^) stained with anti-PD-1 antibody (clone PD1.3.1.3) was normalized to the sample without the addition of therapeutic anti-PD-1 antibodies (prolgolimab, nivolumab and pembrolizumab). Data is shown mean ± SD of results with 3 donors. Statistical analyses were performed on samples after 24 h resting using the two-tailed paired Student’s t-tests.

This sequential binding experiment revealed some differences. Although nivolumab and pembrolizumab almost completely blocked the binding of competing antibodies during their co-incubation with the cells (Supplementary fig. S1), in sequential binding experiments PD-1 was released from complexes with nivolumab or especially pembrolizumab, but not with prolgolimab after incubation for 24 h. The number of cells with unblocked PD-1 approached to 60% for pembrolizumab and 25% for nivolumab, while this did not reach 5% for prolgolimab (Fig. 2). Which correlates with the data of a decrease in the proportion of cells bound to the therapeutic anti-PD-1 antibodies (Supplementary fig. S2).

### Comparison of the functional activities of the anti-PD-1 antibodies

**Fig. 3:**
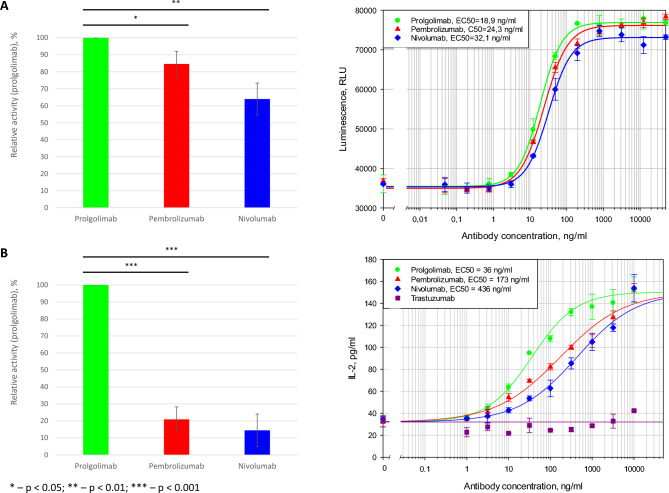
Functional activities of prolgolimab, pembrolizumab and nivolumab. Relative activity was determined according to the EC50 of prolgolimab. (**A**) Reporter bioassay for anti-PD-1 dependent reactivation of NFAT-signaling. (**A, left**) The bar chart with the average relative activities of prolgolimab, pembrolizumab and nivolumab based on the results of 4 experiments. Data are shown as the mean ± SD. Statistical analyses were performed using the two-tailed paired Student’s t-tests. (**A, right**) A representative plot of luminescence intensity versus antibody concentration. (**B**) Bioassay for anti-PD-1 dependent IL-2 secretion by SEB-stimulated PBMCs. (**B, left**) Average relative activities of prolgolimab, pembrolizumab and nivolumab based on the results of 5 experiments. Data are shown as the mean ± SD. Statistical analyses were performed using the two-tailed paired Student’s t-tests. (**B, right**) A representative plot of IL-2 concentrations versus antibody concentration.

Next, we directly evaluated functional consequences of the interaction between therapeutic antibodies and PD-1. The PD-1/PD-L1 interaction suppresses the T-cell-dependent immune response mediated by NFAT signaling. By binding to PD-1, the anti-PD-1 antibody blocks this interaction and, accordingly, causes the reactivation of NFAT signaling. The activities of nivolumab, pembrolizumab and prolgolimab towards NFAT pathways were compared. Prolgolimab demonstrated only marginally higher activity than nivolumab and pembrolizumab in the NFAT reporter test (Fig. 3A).

We next applied an orthologous approach based on PBMCs, as the latter experimental system is composed by primary human cells, which is closer to a real organism. SEB-driven activation of T-cells results in the production of IL-2 by PBMCs, therefore we utilized IL-2 measurement for the comparison of the above antibodies. The activity of prolgolimab in the IL-2 secretion test was approximately 5 times higher than that of pembrolizumab and nivolumab (Fig. 3B).

### Comparison of the affinity of nivolumab, pembrolizumab and prolgolimab for FcγR and FcRn

The antigen-binding portion of therapeutic anti-PD-1 antibodies mediates their clinical activity by binding to PD-1 and thus blocking the interaction of this receptor with PD-L1. At the same time, the Fc part of antibodies can interact with a number of proteins mediating CDC, ADCC, and ADCP.

The affinity of prolgolimab, pembrolizumab and nivolumab to different FcγR molecules was analyzed by bio-layer interferometry. There was no significant difference in the interactions of the aforementioned antibodies with FcRn, FcγRIIa and FcγRIIIa receptors. However, pembrolizumab and nivolumab had many-fold higher affinity to FcγRIa and FcγRIIb than prolgolimab (Table 2).

**Table 2:** Affinity of nivolumab, pembrolizumab and prolgolimab to FcγRs and FcRn. The binding kinetics of anti-PD-1 antibodies to FcγRs and FcRn were determined by biolayer interferometry. Biotinylated receptors were immobilized on Streptavidin (SA) sensor, anti-PD-1 antibodies were in solution.

Receptor	K_D_, M					
	Nivolumab		Pembrolizumab		Prolgolimab	
	Mean	SD	Mean	SD	Mean	SD
FcγRIa	7.4 × 10^–8^	1.91 × 10^–08^	8.50 × 10^–8^	2.43 × 10^–08^	9.00 × 10^–7^	2.12 × 10^–^^07^
FcγRIIa (131R)	2.00 × 10^–6^	1.53 × 10^–08^	5.17 × 10^–6^	7.83 × 10^–07^	9.66 × 10^–6^	4.09 × 10^–07^
FcγRIIa (131H)	1.31 × 10^–5^	4.31 × 10^–07^	1.11 × 10^–5^	8.73 × 10^–07^	1.46 × 10^–5^	1.66 × 10^–07^
FcγRIIb	9.30 × 10^–7^	7.23 × 10^–09^	8.64 × 10^–6^	4.75 × 10^–07^	2.10 × 10^–5^	1.81 × 10^–06^
FcγRIIIa (158V)	8.97 × 10^–6^	4.73 × 10^–07^	5.24 × 10^–6^	1.31 × 10^–07^	1.33 × 10^–6^	5.77 × 10^–08^
FcγRIIIa (158F)	1.66 × 10^–5^	6.18 × 10^–07^	1.99 × 10^–5^	4.81 × 10^–07^	1.27 × 10^–5^	5.77 × 10^–07^
FcRn	2.48 × 10^–8^	1.40 × 10^–10^	2.5 × 10^–8^	1.01 × 10^–10^	1.53 × 10^–8^	6.47 × 10^–10^

### Comparison of the activation of antibody-dependent cellular cytotoxicity (ADCC) and complement-dependent cytotoxicity (CDC) by nivolumab, pembrolizumab and prolgolimab

Antibody-dependent cellular cytotoxicity (ADCC) is mediated by the interaction of FcγRIIIa (CD16) of NK cells with antibodies bound to targets on the cells. The ADCC of anti-PD-1 antibodies could potentially reduce the effectiveness of the antitumor response by eliminating antibody-bound T-cells^22^.

**Fig. 4:**
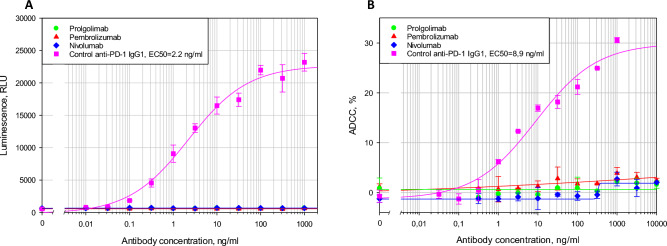
Activation of antibody-dependent cellular cytotoxicity (ADCC) by nivolumab, pembrolizumab and prolgolimab. PBMCs were pre-activated with SEB. (**A**) Evaluation of antibody binding to FcγRIIIa in a reporter bioassay. (**B**) ADCC assessment in the cytotoxicity test with NK cells.

To ascertain that the antibodies cause no ADCC, tests were performed to evaluate the binding to FcγRIIIa in the reporter cell line assay and ADCC itself in the assay using NK cells. In both tests, pre-activated PBMCs containing T-lymphocytes with surface expression of PD-1 were used as target cells (Supplementary fig. S2), an effector antibody (IgG1 with VH and VL of pembrolizumab) was used as a positive control. According to the study results, neither of the three therapeutic antibodies activates CD16-positive cells or causes a cytotoxic effect (Fig. 4). Thus, nivolumab, pembrolizumab, and prolgolimab have no ADCC activity.

Prolgolimab, similarly to nivolumab and pembrolizumab, did not cause CDC (Supplementary Fig. 4).

### Comparison of the activation of antibody-dependent cellular phagocytosis (ADCP) by nivolumab, pembrolizumab and prolgolimab

The ADCP can potentially reduce the antitumor response to anti-PD-1 antibodies due to the elimination of PD-1^+^ T-cells. Therefore, it is important to make sure that these antibodies cannot activate ADCP^22^. ADCP may be mediated by FcγRIa, FcγRIIa, and FcγRIIIa, as all three of them are present on the surface of macrophages.

**Fig. 5:**
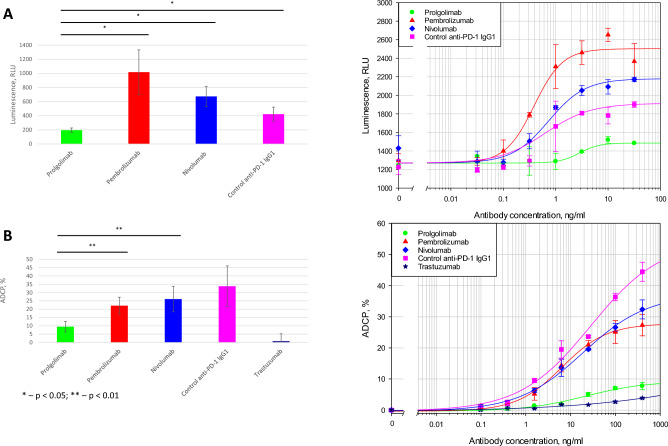
Activation of antibody-dependent cellular phagocytosis (ADCP) by prolgolimab, pembrolizumab and nivolumab. (**A**) Evaluation of the antibody binding to FcγRIa in a reporter bioassay. PBMCs were pre-activated with SEB. (**A, left**) Average differences in luminescence in the presence of 10 ng/ml and in the absence of the test antibodies (based on the results of 3 experiments). Data are shown as the mean ± SD. Statistical analyses were performed using the two-tailed paired Student’s t-tests. (**A, right**) A representative plot of luminescence intensity versus antibody concentration. (**B**) ADCP with monocyte-derived macrophages and PD-1 expressing cells. Negative control—trastuzumab (anti-HER2 antibody). (**B, left**) Average ADCP values in the presence of 400 ng/ml of the test antibodies from the results of 5 experiments. Data are shown as the mean ± SD. Statistical analyses were performed using the two-tailed paired Student’s t-tests. (**B, right**) A representative graph of the proportion of macrophages that phagocytized target cells versus antibody concentration. Control anti-PD-1 IgG1 – IgG1 with pembrolizumab’s variable domains.

First, we evaluated the binding of anti-PD-1 antibodies to FcγRIa and FcγRIIa using a reporter cell line assay. None of the three analyzed antibodies interacted with FcγRIIa (data not shown). In the FcγRIa test, all tested antibodies activated the reporter gene expression. The increase of expression was higher for nivolumab and pembrolizumab (both an IgG4) as compared to prolgolimab and even positive control (the effector antibody – IgG1 with VH and VL of pembrolizumab) (Fig. 5A).

We further analyzed the ability of nivolumab, pembrolizumab, and prolgolimab to induce ADCP using monocyte-derived macrophages and Jurkat cells expressing PD-1. In this case, the ADCP induced by nivolumab and pembrolizumab did not exceed that of the positive control, which is probably due to the contribution to ADCP not only from FcγRIa, but also from FcγRIIa and FcγRIIIa, which have a higher affinity for native IgG1 relative to IgG4. Antibody-dependent phagocytosis is several times less intensive with prolgolimab than with nivolumab and pembrolizumab (Fig. 5B, Supplementary fig. S3).

### Comparative in vivo antitumor activity study of pembrolizumab and prolgolimab

**Fig. 6:**
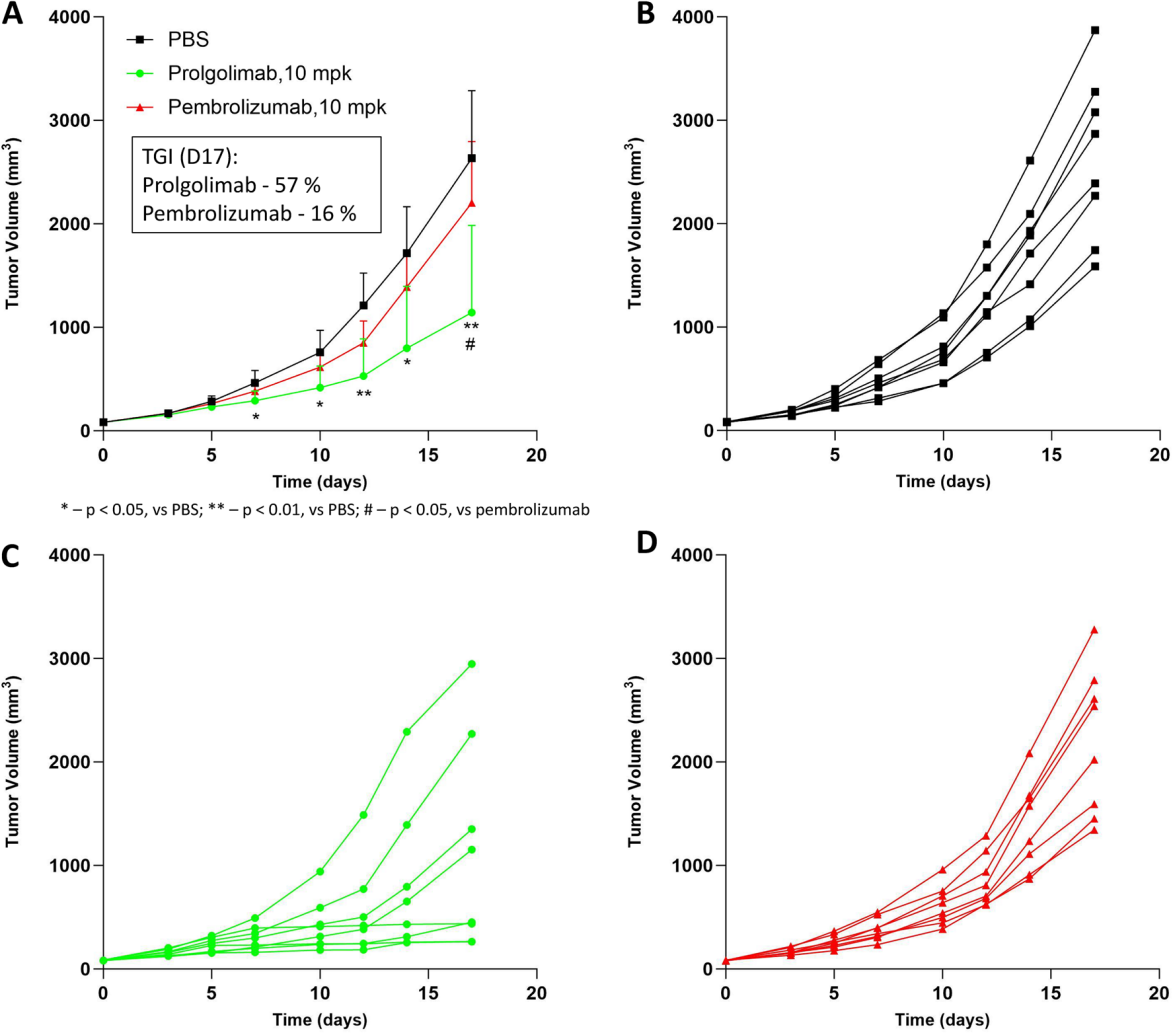
Antitumor effect of pembrolizumab and prolgolimab in vivo**.** The graphs shows the change in the average (**A**) or individual (**B, C, D**) volume of tumors in groups of animals relative to the start time of treatment administration. Error bars—95% confidence interval for mean tumor size. Statistical analyses were performed using the Tukey’s multiple comparisons test. The inset shows tumor growth inhibition index (TGI) data for day 17.

To compare the antitumor activity of pembrolizumab and prolgolimab in vivo, we used a syngeneic model based on transgenic mice of the BALB/c background, in which the sequence of the mouse gene encoding the extracellular part of PD-1 was replaced with the human PD-1 sequence. These mice were inoculated with CT26.wt cells, maintained until the tumor volume reached 80–90 mm^3^, and then treated with pembrolizumab or prolgolimab (Fig. 6). According to tumor volume measurements, the tumor growth was similar in the pembrolizumab and control arms (Fig. 6). At the same time, administration of prolgolimab slowed tumor growth, producing statistically significant differences when compared to pembrolizumab or control. At the last day of the study, the tumor growth inhibition index was 16% for pembrolizumab and 56% for prolgolimab.

## Discussion

This study shows that the IgG1-based LALA-mutated therapeutic antibody prolgolimab may have some potential advantages over the IgG4 antibodies pembrolizumab and nivolumab. In particular, prolgolimab demonstrated more stable interaction with PD-1, induced higher T-cell activation in cell-based bioassays, did not induce ADCP and showed more robust tumor growth inhibition in laboratory animals as compared to the aforementioned drugs.

There has been growing attention to the effector functions of antitumor antibodies and the ways to modify them to increase their antitumor activity. In this study, we used in vitro tests to compare three antitumor monoclonal antibodies against PD-1 receptor, nivolumab (IgG4), pembrolizumab (IgG4) and prolgolimab (IgG1 with the LALA mutation). We evaluated the desirable effects of these antibodies, i.e. their binding to PD-1 aimed at blocking PD-L1/PD-1 binding as well as undesirable properties of these drugs, i.e. the interaction between their Fc regions and FcγRs potentially activating the effector functions of immune cells. IgG4-based monoclonal antibodies are considered to have lower effector functions compared to IgG1. However, there is some evidence in literature regarding the effector functions of these antibodies^11^. At the same time, the LALA mutation eliminates the effector functions of IgG1. However, no direct comparisons of antigen-binding and effector functions of these antibodies have been conducted so far.

The comparison of the antigen-binding properties of the evaluated antibodies revealed no significant differences in the constants of their binding to PD-1. Similarly, no significant differences were found in antibody activity in a reporter test based on the use of immortalized cell lines. Importantly though, the activity of prolgolimab was 5 times higher than that of pembrolizumab or nivolumab in the PBMC test. The discrepancy between the results of the two test systems can be explained by the complexity of the PBMC system that includes a larger number of factors that are difficult to reproduce in the reporter system. The results obtained with PBMCs may be more biologically relevant and, therefore, deserve consideration.

The T-lymphocyte PD-1 receptor occupancy study showed that the binding of prolgolimab to PD-1 is more stable, because the number of cells with unblocked PD-1 was less than 10% at 24 h after removal of prolgolimab, while these values were approximately 20% and 60% for nivolumab and pembrolizumab, respectively (Fig. 2). The occupancy results can be partly explained by the fact that although the three antibodies have similar equilibrium constants (K_D_), the dissociation constant (k_d_) of prolgolimab is slightly lower, which means that the complex of this antibody with PD-1 is potentially more stable. However, the observed advantage in K_D_ for prolgolimab is not high enough to explain such a significant difference in occupancy. The obvious difference of this test is that the measurement of constants by the SPR method is carried out in a simplified "pure" system on sensors, and the PD-1 occupancy test was performed on PBMCs, which is close to a real living system. There is an assumption that myeloid cells upon FcүRI binding can “rob” anti-PD-1 antibodies from T-cells. In this case, the process occurs through dissociation of the PD-1/antibody complex rather than trogocytosis, since PD-1 remains on the surface of T cells. This hypothesis is supported by the direct correlation between the PD-1 occupancy assay (Fig. 2) and the reporter cell-based FcγRI activation assay (Fig. 5A). The reduced interaction of prolgolimab with FcγRI is explained by the nature of the Fc part of the molecule, but this does not explain the differences observed between the two IgG4-based antibodies. It can be hypothesized that the differences are related to the steric accessibility of PD-1-associated nivolumab and pembrolizumab to interact with FcүRI.

Results of the NFAT signaling reactivation reporter test correlate with the obtained K_D_ data, as there is no significant difference between the antibodies. However, prolgolimab has a clear advantage in the more relevant PBMC test, which is consistent with the occupancy data.

A more detailed comparison of the nivolumab epitope with that of PD-L1 shows that only a 14.65% of the PD-L1 epitope surface is covered by this antibody. At the same time, prolgolimab and pembrolizumab cover more than 60% of the PD-L1 epitope. The prolgolimab epitope seems to be more ‘concentrated’ at the PD-L1 epitope, while the pembrolizumab epitope includes much more ‘non-target’ amino acids. Prolgolimab is predicted to interact with highly charged K134 and E135 of PD-1, in contrast to pembrolizumab. Due to their charge property, these amino acids can significantly contribute to PD-1/PD-L1 binding. This assumption is confirmed by molecular simulations of the complex with further assessment of the contribution of these electrostatic interactions to the energy of the complex. Therefore, covering them by antibody potentially prevents this much more efficiently than covering other epitope amino acids.

The interactions of the evaluated antibodies with FcRn, FcγRIIa and FcγRIIIa were found to be similar. The absence of binding antibodies to FcγRIIIa and FcγRIIa was further confirmed in reporter cell assays. The absence of ADCC activity of antibodies mediated by FcγRIIIa of NK cells was shown in the cytotoxicity test.

This study produced highly consistent results in terms of the effector functions of nivolumab, pembrolizumab and prolgolimab. While these antibodies had similarly low affinity for FcγRIIIa and, consequently, did not induce ADCC, nivolumab and pembrolizumab had higher affinity for FcγRIa and FcγRIIb and, in agreement with these findings, showed ability to induce ADCP. These data suggest that when binding to T-cells, prolgolimab does not attract macrophages, and T-cells remain capable of destructing tumor cells.

Differences in the interaction with the FcγRIIb receptor may deserve particular consideration. This receptor is unique in that it is the only FcγR that has inhibitory activity and is capable of exerting an immunosuppressive effect. FcγRIIb has recently been shown to be present on tumor-infiltrating CD8 effector T-cells in a murine melanoma model, as well as on CD8+ T-cells from patients with melanoma. The expression of FcγRIIb on effector CD8 T-cells was associated with the expression of PD-1^23^. Reduced binding of prolgolimab to the FcγRIIb receptor may be of potential value^24–26^. It has been shown that FcγRIIb expressed by lymphoma B-cells can accelerate the internalization of a targeted antibody (anti-CD20 Rituximab) and thus reduce the effectiveness of therapy^27^. Potentially, an anti-PD-1 antibody could similarly be eliminated from the surface of PD-1^+^ cells due to the interaction of the Fc region with FcγRIIb, which could explain the decreasing T-cell receptor occupancy by nivolumab and pembrolizumab. From this point of view, prolgolimab may retain its stability while bound to PD-1 expressing cells, since it has lower interaction with FcγRIIb. The functional role of FcγRIIb in determining differences between pembrolizumab, nivolumab and prolgolimab deserves further evaluation.

The interaction profile of IgG4 and IgG1_LALA with mouse FcgR is comparable to that of human FcgR, making the mouse model suitable for comparison of the corresponding antibodies^28–30^. Because anti-human PD-1 antibodies are not cross-reactive with mouse PD-1, humanized mouse models with transplanted human immune cells are often used for in vivo studies of anti-PD-1 therapy. However, these mice have some defects in the maturation of immune cells and therefore this model has an altered immune system^31^, which, in particular, may compromise the effector functions of antibodies.

A syngeneic model is better suited to reconstitute an adequate immune system, but a model for an anti-human PD-1 antibody must be not just syngeneic, but based on transgenic mice humanized for PD-1. These models are based on BALB/c and C57BL/6 mice. The most commonly used syngeneic model based on B16-F10 melanoma cells^32^ is not sensitive to anti-PD-1 therapy^33^. Another widely used model for testing anti-PD-1 antibodies is the CT26 colorectal cancer cell line model. The cells of this line present on their surface PD-L1, which leads to evasion from the immune system due to PD-1-dependent suppression of T-cell activation. Thus, the CT26-based model is relevant for tumors for which anti-PD-1 therapy is indicated^33^. This model is also interesting because, according to the published studies, this syngeneic mouse model is sensitive to anti-PD-1 therapy in the case of antibodies without effector functions^34^, which are based on rat IgG2a^27^. At the same time, there is evidence of low anti-tumor efficacy of pembrolizumab and nivolumab in this model (BALB/c-Pdcd1^em1(hPDCD1)Asc^, Applied StemCell)^35^. Thus, the CT26 model is suitable to test prolgolimab benefits. Our results obtained in a murine model also confirm the low efficacy of pembrolizumab and demonstrate a significantly higher efficacy of prolgolimab (TGI is 3.5 times higher), which lacks effector functions. This is consistent with and explained by our results of in vitro tests. Therefore, we expect that prolgolimab may perform better than current standards of therapy in clinical use.

## Conclusion

The article presents initial results of preclinical comparative studies of the antigen-binding and effector functions of three anti-PD-1 therapeutic antibodies (nivolumab, pembrolizumab, prolgolimab). Prolgolimab demonstrated higher PD-1 receptor occupancy and higher T-lymphocyte IL-2 secretion compared to nivolumab and pembrolizumab. In addition, none of the evaluated antibodies induced ADCC; however, the ability to activate ADCP was significantly lower for prolgolimab. The obtained evidence of the advantages in PD-1 receptor binding and the reduced effector functions of prolgolimab compared to nivolumab and pembrolizumab may indicate a greater antitumor activity of prolgolimab. This was demonstrated using an appropriate murine model, where prolgolimab demonstrated higher antitumor activity than pembrolizumab. The results of this study warrant clinical comparison of IgG1- and IgG4-based anti-PD-1 antibodies.

## Materials and methods

### in silico* epitope mapping*

The prolgolimab variable domain structure was predicted by in-house pipeline involving the Prime tool from Schrödinger Suite^36^.

The structure of PD-1 crystallized in complex with PD-L1 (PDB ID: 4ZQK) was used as a structure to process. The Protein Preparation Wizard tool of Schrödinger Suite was used to repair the structure^37^. The same processing was applied to the following complexes: PD-1 and nivolumab (PDB ID: 5WT9), PD-1 and pembrolizumab (PDB ID: 5B8C) and PD-1 with PD-L1 (PDB ID: 4ZQK).

Docking of prolgolimab and PD-1 was simulated with the PIPER tool^38^. The docking was set to the specific antibody mode, which adds rewards to the docking scoring function for CDR-involved interactions and penalties for CDR-free interactions. This makes the algorithm select antibody-antigen complex variants with more interactions between CDRs and the target. 30 complex structures after PIPER docking were used for molecular dynamics simulations. Desmond package was used for setting up and conducting these procedures^39^. Molecular dynamics simulations were run using the OPLS3 force field^40^ at 294 K with v-rescale thermostat^41^ and 1 bar pressure maintained with Parrinello-Rahman barostat^42^. Electrostatic interactions were processed using the u-series method^43^. Simulation run time was 10 ns and the timestep was 2 fs. An average MM/GBSA energy was calculated for every complex. The complex with the lowest energy was chosen as a valid one and was used to analyze the epitope of prolgolimab. An epitope for every complex of PD-1 with its ligand or PD-1 binding antibody was determined as a set of PD-1 amino acids within 4 Å of ligand or antibodies. PyMol 2.4.1 package was used to visualize all the complexes, PD-1 surface and its epitopes^44^.

### Surface plasmon resonance (SPR) analysis of binding kinetics between anti-PD-1 antibodies and PD-1 receptor

The kinetics of the binding of anti-PD-1 antibodies to the extracellular domain of PD-1 receptor (R&D Systems) was determined by the SPR method using Reichert4SPR system (Reichert Technologies). Protein from 100 μl of 2 μg/ml PD-1 (ccылкa) solution was immobilized on a Planar Polyethylene Glycol/Carboxyl Sensor Chip (Reichert Technologies) followed by analysis of its binding to anti-PD-1 antibodies serial diluted (10; 3.33; 1.11; 0.37; 0.12 nM) in PBS 0.02% Tween-20 pH 7.4. Multi-step kinetics were performed. The assay was carried out in triplicate. The measurement data were processed using the TraceDrawer software (RidgeView Instruments AB).

### Genetic constructs

The development of the pSX_PD-1 vector expressing PD-1 receptor has been described previously in^45^. Plasmids pSX_CD16 (FcγRIII (158V)), pSX_CD32 (FcγRIIa (131R)) and pSX_CD64 (FcγRIa) were derived from the pSX vector (BIOCAD). The expressed genes in these plasmids are under the control of the CMV promoter. These plasmids also contain an expression cassette with a puromycin resistance gene^45^.

Genetic constructs encoding extracellular fragments of FcγRIa, FcγRIIa (131R), FcγRIIa (131H), FcγRIIb, FcγRIIIa (158V), FcγRIIIa (158F) and FcRn with avi and epea tags on the C-terminal end were made for expression in mammalian cells. Sequences of extracellular fragments of FcγRs and FcRn were assembled according to the Uniprot database.

### Cell cultures

Human venous blood was collected from healthy volunteer donors with their informed consent. All maternal cell lines were obtained from Collection of Vertebrate Cell Cultures (Institute of Cytology, Russian Academy of Sciences). Peripheral blood mononuclear cells (PBMCs) were isolated from heparinized venous blood of donors by Ficoll 1.077 density gradient centrifugation (Ficoll-Paque Premium, GE Healthcare) according to the manufacturer's protocol. The production of Jurkat NFAT-FLuc, Jurkat NFAT-FLuc PD-1 and Raji PD-L1 cell lines is described in^45^.

The Jurkat PD-1 cell line was obtained by electroporation of Jurkat cells with the pSX_PD-1 vector followed by selection in RPMI-1640 medium with 10% FBS containing Geneticin (Life Technologies). Clones expressing PD-1 on the cell surface were selected by cytofluorimetric analysis of cells stained with anti-PD-1 PE antibodies (PD1.3.1.3, Miltenyi Biotec) using Guava 12HT instrument (Millipore).

Jurkat NFAT-FLuc CD16, Jurkat NFAT-FLuc CD32, and Jurkat NFAT-FLuc CD64 cell lines were obtained by electroporation of Jurkat NFAT-FLuc cells with pSX_CD16, pSX_CD32, pSX_CD64 vectors, respectively, followed by selection in RPMI-1640 medium with 10% FBS containing puromycin (Gibco). Clones expressing target receptors on the cell surface were selected by cytofluorimetric analysis of cells stained with anti-CD16 PE (3G8, Biolegend), anti-CD32 PE (FUN-2, Biolegend) or anti-CD64 PE (10.1, Biolegend) antibodies using Guava 12HT (Millipore). The functionality of the clones was confirmed by antibody-dependent activation of luciferase expression in the presence of the antibody target.

### Competition analysis of the diagnostic fluorescent-labeled anti-PD-1 antibody (PD1.3.1.3 clone) and therapeutic anti-PD-1 antibodies (pembrolizumab, nivolumab and prolgolimab) for binding to PD-1 on T-lymphocytes

The PBMC suspension was stained with a panel of antibodies against CD45 PerCP (2D1, Biolegend), CD3 BV510 (SK7, Biolegend) and PD-1 PE (PD1.3.1. 3, Miltenyi Biotec) in the presence of 20 μg/mL of one of the therapeutic anti-PD-1 antibodies. Cytofluorimetric analysis was performed using FACS Canto II (Becton Dickinson). The CD45^+^/CD3^+^ cell population corresponds to T-lymphocytes. The proportion of cells stained with anti-PD-1 antibody (clone PD1.3.1.3) was determined, and the values were normalized to the sample without the addition of therapeutic antibodies.

### Analysis of T-lymphocyte PD-1 receptor occupancy by anti-PD-1 antibodies

Anti-PD-1 antibodies (pembrolizumab, nivolumab or prolgolimab) were added to the PBMC suspensions to a final concentration of 20 μg/mL. After 30 min of incubation at room temperature, the cells were washed and divided into 2 parts: one was used for immediate staining with the antibody panel, the second was incubated in RPMI-1640 with 10% FBS for 24 h at 37 °C with 5% CO_2_, followed by staining with the same antibody panel. The panel of fluorescently labeled antibodies included CD45 PerCP (2D1, Biolegend), CD3 BV510 (SK7, Biolegend), PD-1 PE (PD1.3.1. 3, Miltenyi Biotec) and human IgG Fc APC (Jackson Immunoresearch).

Cytofluorimetric analysis was performed using FACS Canto II (Becton Dickinson). The CD45^+^/CD3^+^ cell population corresponds to T-lymphocytes. The proportion of corresponding cells stained with anti-PD-1 antibody (clone PD1.3.1.3) was determined and normalized to the sample without the addition of therapeutic antibodies, the proportion of PD-1 positive cells in which was taken as 100%. The percent of bound anti-PD-1 therapeutic antibodies represents the ratio of percent of T cells stained with anti-human IgG Fc in samples after resting to the percent of T cells stained with anti-human IgG Fc in the same samples before resting.

### Reactivation of NFAT signaling by anti-PD-1 antibodies in a reporter test

The study was carried out as described in^45^. Each well of a 96-well culture plate contained 50,000 Jurkat NFAT-FLuc PD-1 cells, 25,000 Raji PD-L1 cells, 1 ng/mL of anti-CD3/anti-TAA1 antibodies and serial dilutions of the analyzed anti-PD-1 therapeutic antibodies in 100 µL of RPMI-1640 with 10% FBS. After 16-h incubation at 37 °C with 5% CO_2_, the level of luciferase expression in the wells was assessed using the ONE-Glo kit (Promega), followed by measurement of the luminescence intensity using a SPARK 20M plate reader (Tecan). SigmaPlot software (SYSTAT Software) was used to determine the EC50 of the anti-PD-1 antibodies.

Data calculation and plotting were performed using MS Excel and SigmaPlot software packages. The experimental data of the luminescence intensity on the concentration of the test antibodies were approximated using a four-parametric logistic function.

The relative activity of the drugs was evaluated by comparing the EC_50_ values according to the formula:

$$RA, \%=\frac{{EC}_{50}(test AB)}{{EC}_{50}(prolgolimab)}\times 100\%$$

### Antibody-dependent stimulation of *Staphylococcus* enterotoxin B (SEB) mediated IL-2 secretion by PBMCs

Each well of a 96-well culture plate contained 150,000 PBMCs preactivated by 100 ng/ml SEB for 72 h, 100 ng/mL SEB, and serial dilutions of the analyzed anti-PD-1 therapeutic antibodies in 250 µL of RPMI-1640 with 10% FBS. After incubation for 24 h at 37 °C with 5% CO_2_, the concentration of IL-2 in the culture medium was assessed using the Human IL-2 DuoSet ELISA kit (R&D Systems).

Data calculation and plotting were performed as described in the above paragraph.

### Bio-layer interferometry analysis of the anti-PD-1 to FcγRs and FcRn binding kinetics

Extracellular fragments of FcγRIa, FcγRIIa (131R), FcγRIIa (131H), FcγRIIb, FcγRIIIa (158V), FcγRIIIa (158F) and FcRn were produced in CHO-K1 cell culture cotransfected with the appropriate genetic construct and the genetic construct encoding BirA ligase. Then receptors were purified from cultural supernatants by C‐tag affinity matrix. The efficiency of biotinylation was assessed using Pierce Biotin Quantitation Kit (ThermoScientific).

Measurement of the kinetics of the interaction of the antibodies with receptors FcγRIa, FcγRIIa(131H), FcγRIIa(131R), FcγRIIb, FcγRIIIa(158V), FcγRIIIa(158F) and FcRn was carried out on a ForteBio Octet Red384 device (Sartorius) using biolayer interferometry.

Receptors from 85 μl of 5 μg/ml FcγRIIa (131R), FcγRIIa (131H), FcγRIIb, FcγRIIIa (158V), FcRn or 15 μg/ml FcγRIa solutions were immobilized on Streptavidin (SA) Biosensors (Sartorius followed by analysis of its binding to anti-PD-1 antibodies serial diluted (5520, 2760, 1380, 690, 345, 172.5, 86.25 nM for FcRIa; 34480, 17240, 8620, 4310, 2155, 1078, 538.8 nM for FcRIIa131R, FcRIIa131H; 41380, 20690, 10345, 5173, 2586, 1293.1, 646.6 nM for FcRIIb; 10340, 5170, 2585, 1292.5, 646.3, 323.13, 161.56 nM for FcRIIIa158V; 27590, 13795, 6898, 3448.8, 1724.38, 862.188, 431.093 nM for FcRIIIa158F; and 345, 172.5, 86.25, 43.125, 21.563, 10.781, 5.39 nM for FcRn) in PBS buffer pH 7.4 0.1% BSA 0.1% Tween-20 for FcγRs or in PBS-HCl pH 6.0 0.1% Tween-20 for FcRn. The assays were carried out at least in triplicate. The measurement data were processed using Octet Data Analysis 9.0 software.

#### Complement-dependent cytotoxicity (CDC)

Each well of a 96-well culture plate contained 50,000 Jurkat PD-1 cells and serial dilutions of the test antibodies in 150 µL of RPMI-1640 with 0.1% BSA and freshly thawed 8,3% complement solution (Quidele). After 2-h incubation at 37 °C 5% CO_2_, 15 µL per well of alamarBlue Cell Viability Reagent (Thermo Scientific) was added. After 20-h incubation at 37 °C 5% CO_2_, fluorescence intensity was measured at 544/590 nm using an Infinite M200Pro plate reader (Tecan).

#### Assessment of the binding of anti-PD-1 antibodies to FcγRs in a reporter cell assay

Each well of a 96-well culture plate contained 25,000 Jurkat NFAT-FLuc CD16 or Jurkat NFAT-FLuc CD32 or Jurkat NFAT-FLuc CD64 cells, 50,000 PBMCs preactivated by 100 ng/ml SEB for 72 h, and serial dilutions of the analyzed anti-PD-1 antibodies in 100 µL of RPMI-1640 with 10% FBS. After 16-h incubation at 37 °C with 5% CO_2_, the level of luciferase expression in the wells was assessed using the ONE-Glo kit (Promega), followed by measurement of the luminescence intensity using a SPARK 20M plate reader (Tecan). SigmaPlot software was used to determine the EC50 of the anti-PD-1 antibodies.

#### Antibody-dependent cellular cytotoxicity (ADCC) on NK cells

Each well of a 96-well culture plate contained 50,000 PBMCs preactivated by 100 ng/ml SEB for 72 h, 40,000 NK cells isolated from PBMCs by NK cell Isolation Kit human (Miltenyi Biotec), and serial dilutions of the test antibodies in 150 µL of RPMI-1640 with 10% FBS. After 16-h incubation at 37 °C with 5% CO_2_, the degree of cell lysis was assessed using a CytoTox96®Non-Radio Cytotoxicity Assay kit (Promega), followed by absorbance measurement at 490 nm using a Spark 20M plate reader.

Data calculation and plotting were performed using MS Excel and SigmaPlot software packages. The experimental data of the ADCC dependence on the concentration of the test antibodies were approximated using a four-parametric logistic function.

The effectiveness of ADCC was assessed by the formula:

$$ADCC, \, \% = \frac{{OD\;\left( {Experimental\;data} \right)\; - \;OD\;\left( {TC + NK} \right)}}{{OD\;\left( {TC + lys} \right) - OD\;\left( {TC + NK} \right)}} \times {1}00\% ,$$

where, TC—target cells (SEB preactivated PBMCs); OD (Experimental data) – absorbance in the variants containing the TC, NK cells and the corresponding amount of antibodies; OD (TC + NK)—mean absorbance of the variants containing the TC, NK cells (without antibodies); OD (TC + lys)—mean absorbance of the variants containing TC lysates.

#### Antibody-dependent cellular phagocytosis (ADCP)

PBMCs were isolated from heparinized venous blood of healthy donors by Ficoll density gradient centrifugation. The cells were enriched with monocytes by negative selection using a Classical Monocyte Isolation Kit, human (Miltenyi Biotec GmbH). M0 macrophages were obtained by culturing monocytes in X-VIVO 15 medium (Lonza) supplemented with M-CSF up to 100 ng/mL (Sigma) in flasks with Ultra-Low Attachment surface, at 37 °C with 5% CO_2_ for 6 days. Differentiation into M2c macrophages was performed by adding IL-10 (Peprotech) up to 20 ng/mL, followed by cell incubation at 37 °C with 5% CO_2_ for 24 h.

Jurkat PD-1 cells were stained with the fluorescent dye Calcein AM (Santa Cruz Biotech). Each well of a 96-well culture plate with Ultra-Low Attachment surface contained 100 µL of X-VIVO 15 with 10,000 macrophages, 60,000 Jurkat PD-1 cells stained with Calcein AM, and serial dilutions of the anti-PD-1 antibodies. After 2-h incubation at 37 °C with 5% CO_2_, the cells were stained with anti-CD11b antibodies (ICRF44, Biolegend). Cytofluorimetric analysis was performed using Guava 12HT. In data analysis, ADCP was defined as the proportion of cells positive for Calcein AM and CD11b staining to all CD11b^+^ cells. The ADCP values in the samples without the addition of antibodies were subtracted from the ADCP values of the samples as background.

#### Comparative in vivo antitumor activity study of pembrolizumab and prolgolimab

In vivo studies were carried out at GemPharmatech Co. Ltd., China. Female BALB/cJGpt—*Pdcd1*^*em1Cin(hPDCD1)*^* Tnfrsf18*^*tm1(hTNFRSF18)*^*/* Gpt mice were inoculated with 5 × 10^5^ CT26.wt murine colorectal carcinoma cells subcutaneously in the right flank. When the average tumor volume reached 80–90 mm^3^, the animals were divided into 3 groups of 8 mice each, so that their tumors were comparable between groups in size. Pembrolizumab or prolgolimab were administered at a dose of 10 mg/kg intraperitoneally at days 0, 3, 7, 10, 14 after group formation. During the study, the size of the tumors was measured with a caliper at days 0, 3, 5, 7, 10, 12, 14 and 17 from the start of drug administration. Tumor volumes were determined by the following formula:

$$volume=\frac{{width}^{2}\times length}{2}$$

Tumor growth inhibition (TGI) index was determined by the following formula:

$$TGI=\Big[1-\frac{mean\left({RTV}_{treated}\right)}{mean\left({RTV}_{vehicle}\right)}\Big]\times 100\%,$$

where, RTV—relative tumor volume. RTV was determined as:

$$RTV = \frac{{V_t}}{{V_0}},$$

where, V_0_—tumor volume at the day of the first administration; V_t_—tumor volume at day *t* after antibody administration.

The protocol and any amendments or procedures involving the care and use of animals in this study were reviewed and approved (Animal Protocol No. GPTAP20201230-2) by the Institutional Animal Care and Use Committee (IACUC) of GemPharmatech Co., Ltd prior to conduct. During the study, the care and use of animals were conducted in accordance with the regulations of the Association for Assessment and Accreditation of Laboratory Animal Care (AAALAC), which is consistent with the ARRIVE guidelines.

## Supplementary Information

Supplementary Information.

## Data Availability

The data that support the findings of this study are available in Figs. 1–6, Tables 1–2 and the Supporting Information of this article.
