# Author Correction: Preclinical comparison of prolgolimab, pembrolizumab and nivolumab

**DOI:** 10.1038/s41598-024-77480-w

**Published:** 2024-11-06

**Authors:** Aleksandr Gordeev, Andrei Vaal, Maria Puchkova, Iana Smirnova, Aleksandr Doronin, Anna Znobishcheva, Daria Zhmudanova, Aleksei Aleksandrov, Mikhail Sukchev, Evgeny Imyanitov, Valery Solovyev, Pavel Iakovlev

**Affiliations:** 1 JSC BIOCAD, St.-Petersburg, Russia; 2 Department of Tumor Growth Biology, N.N. Petrov Institute of Oncology, St.-Petersburg, Russia

Correction to: *Scientifc Reports* 10.1038/s41598-024-72118-3, published online 04 October 2024

The original version of this Article contained errors in the names of the authors Aleksandr Gordeev, Andrei Vaal, Maria Puchkova, Iana Smirnova, Aleksandr Doronin, Anna Znobishcheva, Daria Zhmudanova, Aleksei Aleksandrov, Mikhail Sukchev, Evgeny Imyanitov, Valery Solovyev & Pavel Iakovlev, which were incorrectly given as Gordeev Aleksandr, Vaal Andrei, Puchkova Maria, Smirnova Iana, Doronin Aleksandr, Znobishcheva Anna, Zhmudanova Daria, Aleksandrov Aleksei, Sukchev Mikhail, Imyanitov Evgeny, Solovyev Valery & Iakovlev Pavel.

The original Article has been corrected.

