# Supplementary material for: Preclinical comparison of prolgolimab, pembrolizumab and nivolumab
Source: Sci Rep. 2024 Oct 4;14:23136. doi: 10.1038/s41598-024-72118-3 (PMC11452378; doi:10.1038/s41598-024-72118-3)
Supplement: Supplementary file 1 — Supplementary Information. [file 41598_2024_72118_MOESM1_ESM.pdf]

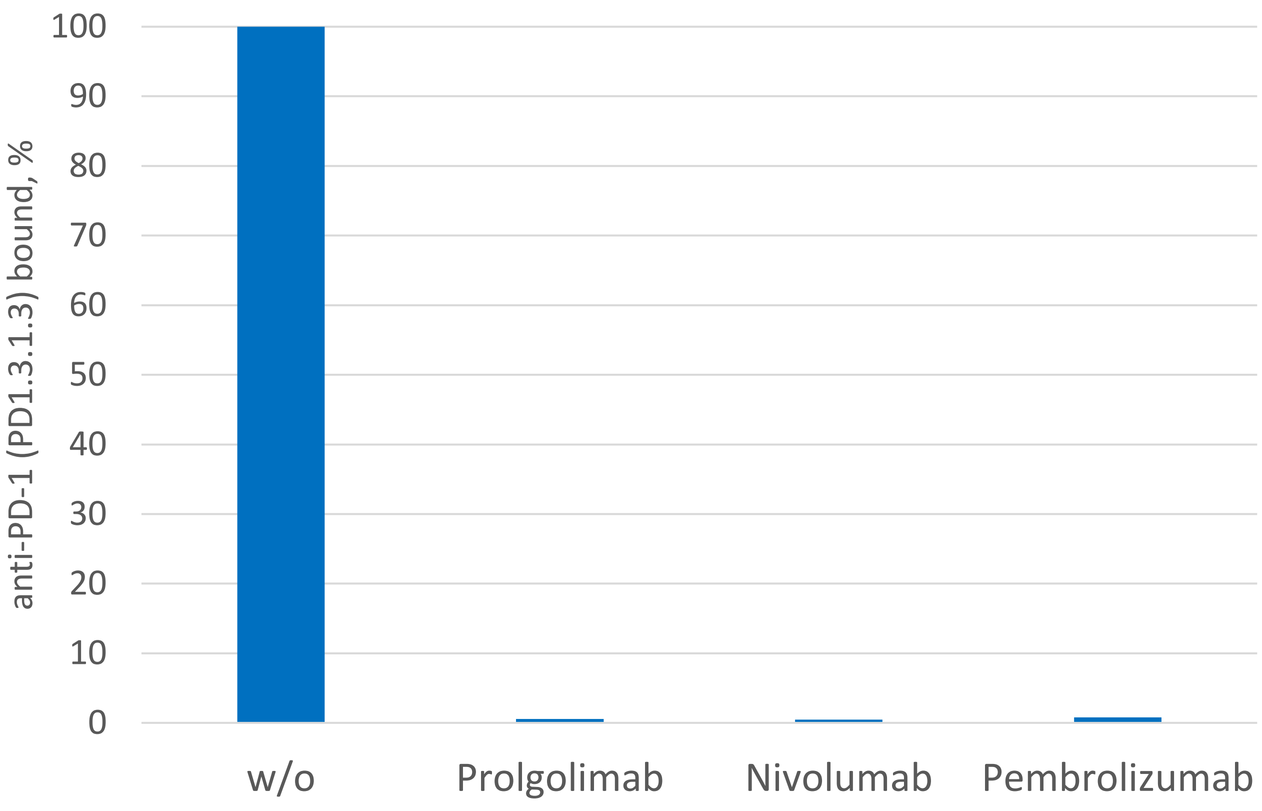


**Supplementary figure S1.**

**Concurrent binding of nivolumab, pembrolizumab and prolgolimab with anti-PD-1 antibody (PD1.3.1.3) for binding to PD-1 receptor of T-cells.**

PBMCs were stained with anti-PD-1 antibodies (clone PD1.3.1.3) in the presence of prolgolimab, nivolumab or pembrolizumab. The proportion of T-lymphocytes ($CD45^+$/$CD3^+$) stained with anti-PD-1 antibody (clone PD1.3.1.3) was normalized to a sample without the addition of nivolumab, pembrolizumab and prolgolimab (w/o).

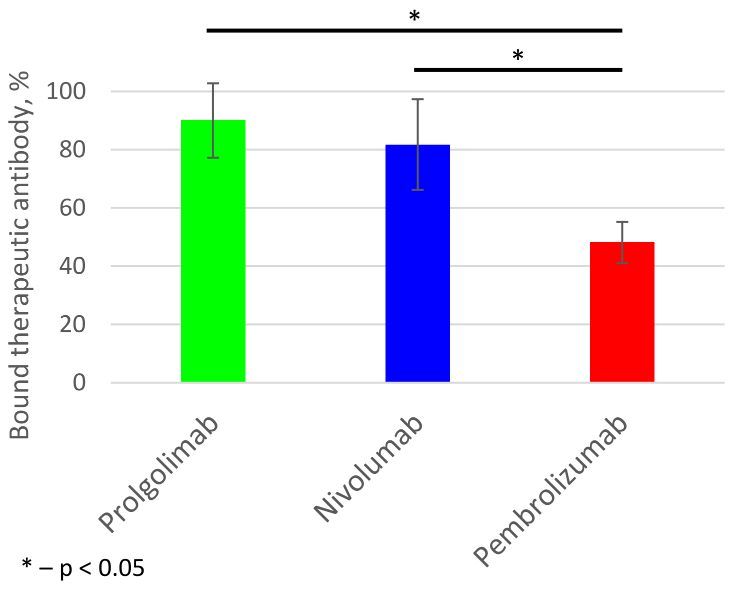


**Supplementary figure S2. Binding of prolgolimumab, nivolumab and pembrolizumab to PBMCs after 24-hour resting.** PBMCs were preincubated with prolgolimab, nivolumab and pembrolizumab, then unbound antibodies were washed off. One portion of the cells was stained with the antibody panel immediately (0h), while the remaining portion was stained after 24 hours of the incubation (24h) in the culture medium. The percent of bound anti-PD-1 therapeutic antibodies represents the ratio of percent of T cells stained with anti-human IgG Fc in samples after resting to the percent of T cells stained with anti-human IgG Fc in the same samples before resting. Data is shown mean ± SD of results with 3 donors. Statistical analyses were performed on samples after 24 hours resting using the two-tailed paired Student's t-tests.

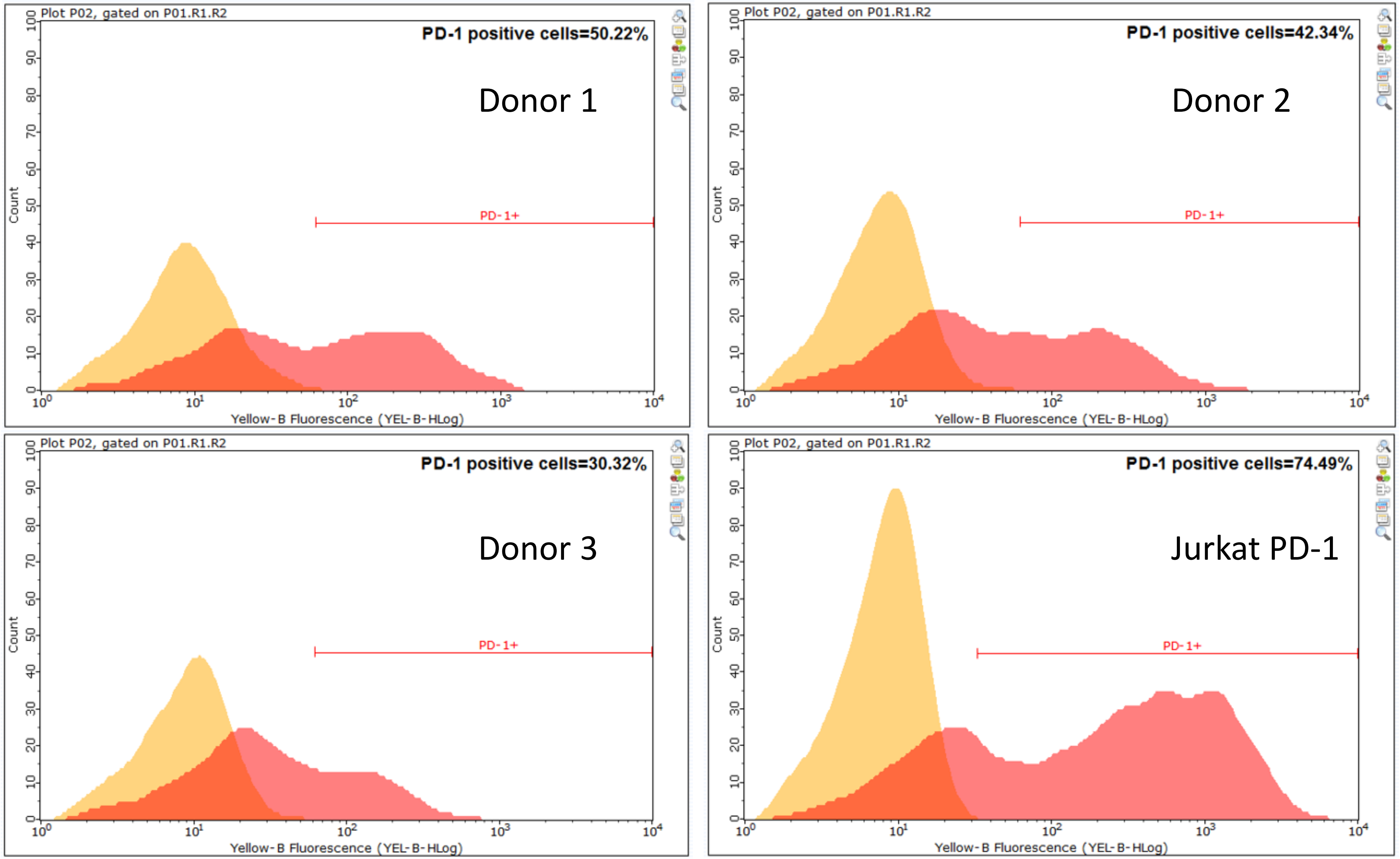


**Supplementary figure S3.**

**PD-1 expression on PBMCs preactivated by SEB for 72 hours (3 donors) and on Jurkat PD-1 cell line.**

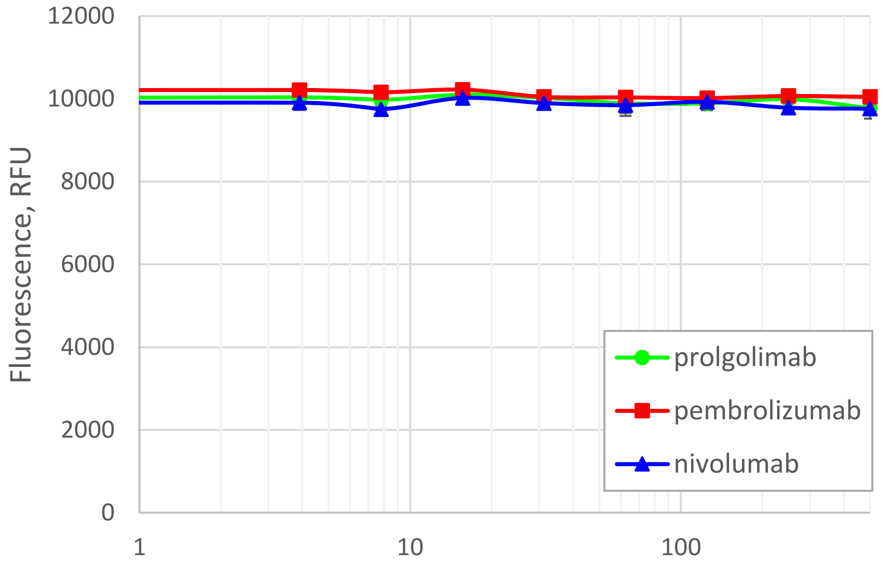


**Supplementary figure S4. Prolgolimab, nivolumab ang pembrolizumab do not cause complement-dependent cytotoxicity (CDC).**

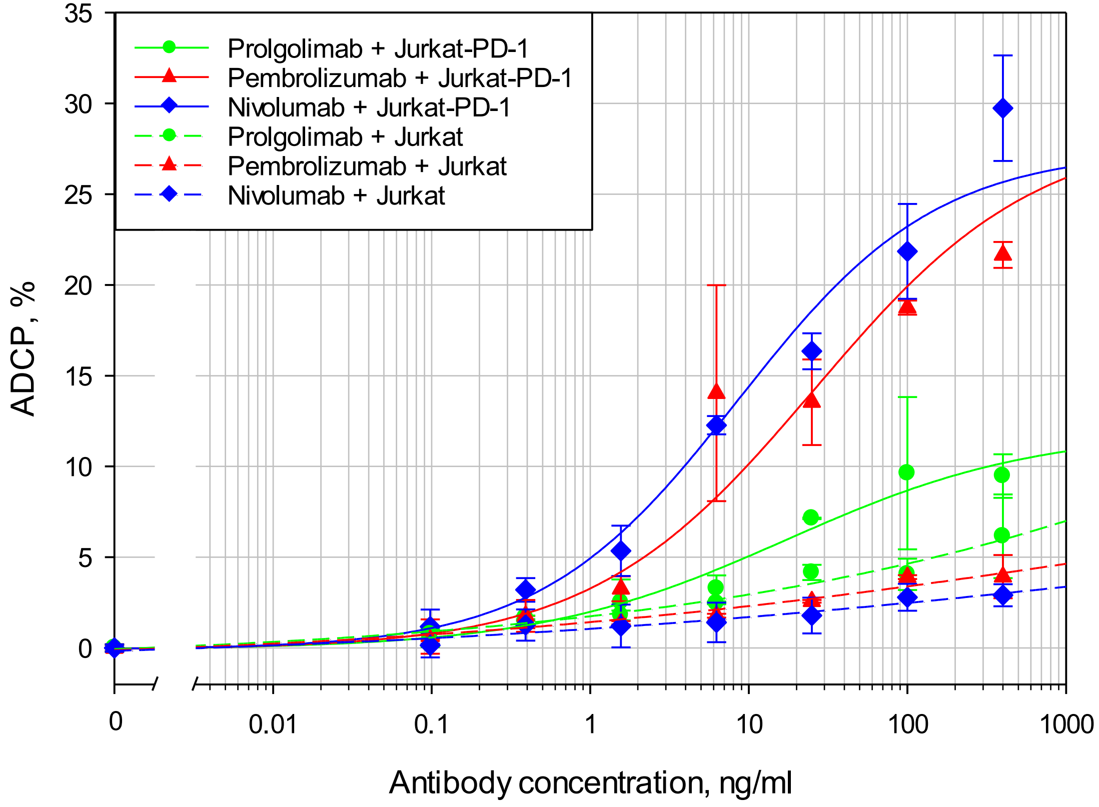


**Supplementary figure S5.**

**Activation of antibody-dependent cellular phagocytosis (ADCP) by nivolumab, pembrolizumab and prolgolimab on PD-1 expressing and non-expressing cells.**
